# Non fourier heat transfer enhancement in power law fluid with mono and hybrid nanoparticles

**DOI:** 10.1038/s41598-021-00423-2

**Published:** 2021-10-22

**Authors:** M. Adil Sadiq

**Affiliations:** 1 Department of Mathematics, DCC-KFUPM, Box 5084, Dhahran, 31261 Saudi Arabia; 2 Interdisciplinary Research Center for Hydrogen and Energy Storage, Dhahran, 31261 Saudi Arabia

**Keywords:** Mathematics and computing, Applied mathematics

## Abstract

Several polymers like ethylene glycol exhibit non-Newtonian rheological behavior. Ethylene glycol is a world-widely used engine coolant and therefore, investigation of thermal enhancement by dispersing mono and hybrid nanoparticles in ethylene glycol is worthful. Since ethylene glycol has shear rate-dependent viscosity and it obeys the power-law rheological model. Therefore, based on these facts, the power-law rheological model with thermophysical properties is augmented with basic law of heat transfer in fluid for the modeling of the considered physical situation. $Mo{S}_{2}$ are taken as mono-nanoparticles where $Mo{S}_{2}$ and $Si{O}_{2}$ are taken as hybrid nanoparticles. Comparative study for the enhancement of thermal performance of MoS2 ethylene glycol and $Mo{S}_{2}$−$Si{O}_{2}$– ethylene glycol is done. For energy conservation, non-Fourier’s law of Cattaneo–Christov is used. The power-law fluid becomes more heat generative due to the dispersion of $Mo{S}_{2}$ and $Si{O}_{2}$. However, $Mo{S}_{2}$−power-law fluid is less heat generative relative to $Mo{S}_{2}$− $Si{O}_{2}$-nanofluid. Thermal relaxation time is found proportional to the ability of the fluid to restore its thermal equilibrium.

## Introduction

Natural and industrial fluids show deviation from Newton's law of viscosity because their viscosity does not show linear relation with the rate of deformation. Such fluids are called non-Newtonian fluids. Non-Newtonian fluids are further classified into many classes. Shear rate-dependent non-Newtonian fluids are a well-known class of non-Newtonian fluids. Fluids of shear rate-dependent viscosity are further classified into power-law fluid^1^, Carreau fluids^2^, Carreau-Yasuda fluid^3^, Bingham fluids^4^, Casson fluids^5^, Herschel-Bulkley^6^, etc. The power-law fluid model is used here in this study because it best describes the rheological behavior of ethylene glycol^7^ as the objective of this study is to discuss the thermal performance of ethylene glycol. In reference^7^, it is claimed that the rheological behavior of ethylene glycol is characterized by a constitutive equation called the power law model. Therefore, the power law constitutive model is selected for the present study. Further, ethylene glycol is electrically conducting and remarkable Lorentz force arises due to the movement of ethylene glycol when it is subjected to the magnetic field. Moreover, ethylene glycol is used as a car engine coolant because it has anti-freezing and anti-boiling properties that encounter heat transfer. This is the main reason for studying heat transfer enhancement in ethylene glycol.

The reason for the selection of the power-law model for the rheology of ethylene glycol is because of recent work by Minako et al.^7^. Although this rheological model has been used in several studies but here, we discuss only those which related to the present investigation. For example, Nawaz et al.^8^ discussed two-dimensional heat transfer enhancement in the power-law fluid by considering hybrid nanofluid. Cheng^9^ discussed simultaneous transport of heat and mass in power-law fluid subjected to mass and thermal stratification over a vertical wavy surface. Khan et al.^10^ performed theoretical analysis for heat and mass transfer in power-law fluid over a surface subjected to convective boundary conditions. Sarafan et al.^11^ examined heat and mass transfer in power-law fluid in a microchannel immersed in a porous medium. Pal and Chatterjee^12^ analyzed Soret and Dufour effects on heat and mass transfer in power fluid over a vertical surface subjected to Buoyancy force considering variable thermal conductivity. El-Kabeir et al.^13^ discussed the impact of stagnation point flow on heat and mass transfer in power-law fluid over moveable walls with the consideration of combined effects of Soret and Dufour and chemical reaction. However, it is very important to mention that studies^9–13^ described above have not considered ethylene glycol as a power-law fluid. This arises an anomaly with recently published work^7^ proving ethylene glycol as a power-law rheological fluid. In view of this published fact, the present study considered the enhancement of heat transfer in ethylene glycol.

Heat transfer has its importance as it occurs in many natural and man-made processes. Heat exchangers, thermal and cooling systems, energy storage, solar systems, MHD generators, food processing processes, etc. are well-known processes where heat transfer is an integral part. The studies related to heat transfer are numerous. However, here, we describe the investigations which are mostly related to the present work. For example, Dogonchi and Ganji^14^ studied the combined impact of thermal radiation, buoyancy force, and nanoparticles on heat transfer in the fluid under Brownian motion. Dogonchi et al.^15^ discussed the impact of viscous dissipation and dispersion of nanoparticles on heat transfer in fluid with copper nanoparticles filled in a permeable enclosure. Sheikholeslami et al.^16^ used a numerical method to discuss the role of nanoparticles on heat transfer in an electrically conducting fluid subjected to the magnetic field and viscous dissipation. Sheikholeslami and Sadoughi^17^ analyzed heat transfer in fluid filled in a cavity with pores. They considered nanoparticles in the fluid and discussed the influence of nanoparticles on heat transfer in MHD fluid using the mesoscopic method. Qureshi et al.^18^ used the finite element method to analyze the impact of nanoparticles on heat transfer in the fluid passing through a porous medium. Nawaz et al.^19^ discussed the Soret and Dufour effects on simultaneous transport of mass heat transfer in MHD flow of viscous fluid over a radially moving surface. Hayat et al.^20^ studied heat transfer in micropolar fluid in the presence of homogeneous-heterogeneous chemical reactions. They considered the above-mentioned effects in fluids over a curved surface.

It is also observed that the studies^14–20^ and the references therein are restricted to the classical Fourier law of heat conduction which does not measure heat conduction in shear rate dependent fluid (like power-law fluid) accurately because classical law of heat conduction does not give a prediction of thermal relaxation time. This limitation was fixed by Cattaneo^21^ and Christov^22^ and the modified law of Fourier is called generalized Fourier law or Cattaneo-Christov heat flux model. This generalized law has been used in various studies. However, no study on thermal enhancement in ethylene glycol treating as the power-law fluid has been discussed so far.

The efficiency of the process related to heat transfer can be enhanced by using a working fluid with higher thermal conductivity. The thermal conductivity of the working fluid can be enhanced by the dispersion of nanoparticles on the fluid. This dispersion of nanoparticles is possible now as synthesis and dispersion of such nanoparticles in a base fluid can be made due to the advancement in technology. Having this fact in mind, researchers have analyzed the role of nanoparticles on thermal enhancement. For example, Nawaz et al.^23^ discussed the role of suspension of nanoparticles on an enhancement in partially ionized fluid during mass transport of species. Qureshi et al.^18^ studied the role of suspension of nanoparticles on heat transfer in magnetohydrodynamic flow exposed to the magnetic field of constant intensity in the presence of mass transfer and chemical reaction. Ellahi et al.^24^ examined the role of nanoparticles on heat transfer in blood moving under the peristaltic mechanism in a couple of stress-fluid. Sheikholeslami et al.^25^ visualized the impact of dispersion of nanoparticles on heat transfer in MHD fluid moving in a porous medium. Sandeep and Kumar^26^ considered suspension of nanoparticles in fluid flow subjected to the simultaneous transport of heat and mass. Qi et al.^27^ modeled heat transfer in the fluid under the influence of suspension of nanoparticles and solved the problems using the Lattice Boltzmann approach to visualize thermal enhancement in the fluid. Gheynani et al.^28^ examined the effect of the size of nanoparticles on heat transfer in non-Newtonian fluid flow. They considered carboxymethyl cellulose as a base fluid. Archana et al.^29^ modeled heat transfer enhancement in yield stress exhibiting fluid (Casson fluid) under the effects of the suspension of nanoparticles and time dependent magnetic field. They solved the problems numerically and investigated the role of various factors on heat transfer in the fluid. They recommended the use of nanoparticles in the working fluid for the improvement in the efficiency of thermal systems.

As far as thermal enhancement of heat transfer is concerned, the dispersion of hybrid nanoparticles (nanoparticles of more than one kind) is recommended as the dispersion of hybrid nanoparticles results in an optimized enhancement in heat transfer. The recent works on hybrid nanofluids are described here. For example, Ahmad et al.^30^ discussed the impact of simultaneous dispersion of copper and aluminum oxide on heat transfer and mass transport in the fluid passing through a porous medium. Ghadikol et al.^31^ considered the dispersion of $Ti{O}_{2}$ and $CuO$ in the mixture of ethylene glycol and water and analyzed their impact on heat transfer enhancement. Alharbi^32^ and Ramesh^33^ performed numerical simulations related to the enhancement of heat transfer in the fluid. Hossein et al.^34^ noted the optimized heat transfer in fluid containing hybrid nanoparticles. Sreedevi et al.^35^ and Alharbi et al.^36^ recommended the dispersion of hybrid nanoparticles for the optimized enhancement in thermal transport in the fluid. Several authors have studied the impact of hybrid nanoparticles $M{oS}_{2}$ and $Si{O}_{2}$ in ethylene glycol but they have treated ethylene glycol either Newtonian fluid or non-Newtonian fluid other than power-law fluid which is according to references^7^, not per recent development in the field. To fill this gap, authors have considered dispersion of $M{oS}_{2}$ and $Si{O}_{2}$ in ethylene glycol and modeled the thermal enhancement in ethylene glycol by treating it to obey power-law rheological behavior.

Reddy et al.^37^ considered the role of hybrid nanoparticles on the enhancement of heat transfer in the MHD flow of fluid over a rotating disk exposed to the magnetic field. They solved related models numerically and noted from simulations that an optimized enhancement of heat transfer in a fluid is possible by the dispersion of hybrid nano-sized particles of higher thermal conductivity. Kumar et al.^38^ considered the simultaneous effects of hybrid nanoparticles and thermal radiations on the heat transfer enhancement in the fluid. They obtained coupled models and solved them numerically to investigate the phenomenon. They noted maximized heat transfer due to the dispersion of hybrid nanoparticles in the fluid. They concluded that working fluid with hybrid nanoparticles is a better coolant and an optimized transport of heat is possible. Reddy et al.^39^ used novel heat flux theory to model heat transfer enhancement in the fluid and examined the role of hybrid nanoparticles on heat transfer subjected to the thermal relaxation time characteristics. Kumar et al.^40^ numerically discussed the impact of hybrid nanoparticles on thermal enhancement in the radiative fluid occupying space in a moving frame of reference. They also analyzed the impact of the spherical shapes of nanoparticles on the heat transport in the fluid. Bai et al.^41^ analyzed the combined effects of stagnation point flow and thermal relaxation time on heat transfer in the Oldroyd-B fluid. Similar studies related to Cattaneo-Christov can be seen in the references^42,43^.

Authors through a literature survey knew that no study on three-dimensional heat transfer enhancement in power-law fluid (ethylene glycol) due to the simultaneous dispersion of $$Mo{S}_{2}$$ and $$Si{O}_{2}$$ has been investigated yet. It is noticed that ethylene glycol has been considered by several researchers but no one has considered ethylene glycol as a power-law fluid. Secondly, no one has studied the effects of hybrid nanoparticles ($$Mo{S}_{2}$$ and $$Si{O}_{2})$$ on heat transfer in ethylene glycol treating it power-law fluid. Moreover, no study on thermal enhancement in a power-law fluid using non-Fourier law of heat conduction is studied yet and no study on three dimensional heat transfers in ethylene glycol as power-law fluid over the two-dimensional nonlinearly stretching surface has been conducted so far. Further, such problems for heat transfer enhancement as modeled in this case have not been solved by the finite element method (FEM). This investigation covers these aspects mentioned above. This investigation consists of five sections. Section one is the background of this problem. Models and their formulation is given in section two. The numerical method is discussed in section three. Outcomes are described in section four. The key outcomes are listed in section five.

## Modeling and models

Power-law rheological models are capable of exhibiting shear rate-dependent viscosity. Several industrial products during the thermal process behave as shear rate-dependent fluid. Modern research has revealed that fluid with shear rate-dependent viscosity, does not obey the classical law of heat conduction. Therefore, we model transfer in the power-law fluid using the non-classical law of heat conduction. This non-Fourier law of heat conduction was proposed by Cattaneo^21^ and Christov^22^ which for heat transfer in incompressible flow is given by

$${\varvec{q}}+{\lambda }_{1} \left[\frac{\partial q}{\partial t}+V\cdot \nabla q+\left(\nabla \cdot V\right)q\right]=-k\nabla T,$$

where ${\varvec{q}}$ is the heat and mass flux.

Thus, governing laws and power-law constitutive models are simultaneously used for the development of the problem. The power-law constitutive equation is given by^44^

1 $${\tau }_{xz}=K{\left|\frac{\partial u}{\partial z}\right|}^{n-1}\frac{\partial u}{\partial z}$$

2 $${\tau }_{yz}=K{\left|\frac{\partial v}{\partial z}\right|}^{n-1}\frac{\partial v}{\partial z}$$

## Problems statement

**Figure 1:**
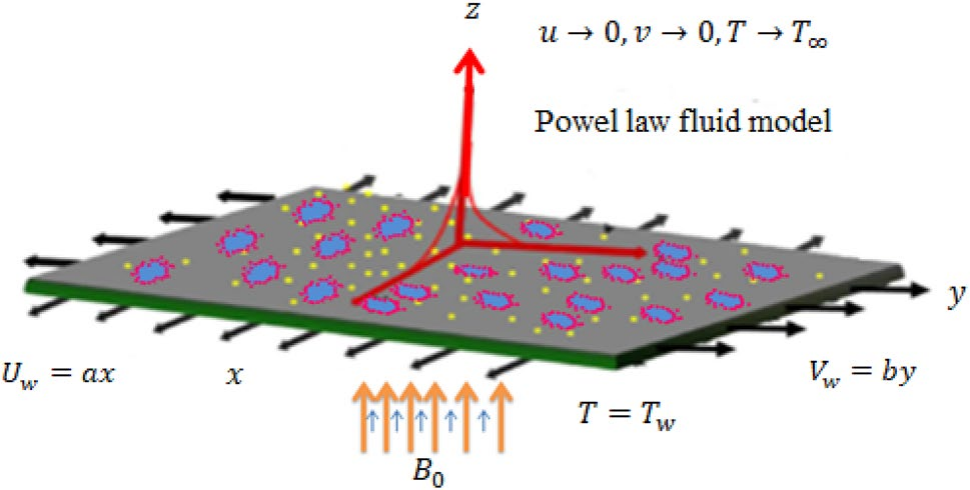
Physical model with coordinate representation.

Let us consider a two-dimensionally moving stretchable surface having a constant temperature ${T}_{w}$. The surface is moving with velocity ${V}_{w}=ax\widehat{\text{i} }+by\widehat{j}$ where $a$ and $b$ are constants having a unit ${s}^{-1}$. Fluid over a surface is non-Newtonian and obeys the power-law rheological model describing shear rate-dependent viscosity. The heat transfer process is assumed to be enhanced using nanoparticles of one kind $(Mo{S}_{2})$ and hybrid nanoparticles (combination of $Mo{S}_{2}$ and $\mathit{Si}{O}_{2}$). It is aimed to compare the enhancement in heat transfer in $Mo{S}_{2}$-power-law fluid and $Mo{S}_{2}-\mathit{Si}{O}_{2}$-power-law fluid. Moreover, ethylene glycol is taken as a base fluid. Comparative analysis among both types of mixtures ($Mo{S}_{2}$-power-law fluid and $Mo{S}_{2}-\mathit{Si}{O}_{2}$-power-law fluid) based on graphical and numerical outcomes will be done. As the wall is moving with two-dimensional velocity, therefore the flow of fluid and heat transfer will be three-dimensional. The geometry of the problem is described in Fig. 1. The conservation laws will be approximated by the boundary layer approximations. Hence, approximated 3D equations are given by^43^

3 $$\frac{\partial u}{\partial x}+\frac{\partial v}{\partial y}+\frac{\partial w}{\partial z}=0,$$

4 $$u\frac{\partial u}{\partial x}+v\frac{\partial u}{\partial y}+w\frac{\partial u}{\partial y}=\frac{{k}_{1}}{{\rho }_{hnf}}\frac{\partial }{\partial z}\left({\left|\frac{\partial u}{\partial z}\right|}^{n-1}\frac{\partial u}{\partial z}\right)-\frac{{B}_{o}^{2}{\sigma }_{hnf}}{{\rho }_{hnf}}u,$$

5 $$u\frac{\partial v}{\partial x}+v\frac{\partial v}{\partial y}+w\frac{\partial v}{\partial y}=\frac{{k}_{1}}{{\rho }_{hnf}}\frac{\partial }{\partial z}\left({\left|\frac{\partial u}{\partial z}\right|}^{n-1}\frac{\partial v}{\partial z}\right)-\frac{{B}_{o}^{2}{\sigma }_{hnf}}{{\rho }_{hnf}}v,$$

$$u\frac{\partial T}{\partial x}+v\frac{\partial T}{\partial y}+w\frac{\partial T}{\partial z}={\lambda }_{1}\left[{u}^{2}\frac{{\partial }^{2}T}{\partial {x}^{2}}+{v}^{2}\frac{{\partial }^{2}T}{\partial {y}^{2}}+{w}^{2}\frac{{\partial }^{2}T}{\partial {z}^{2}}\right]+{\lambda }_{1}\left(u\frac{\partial u}{\partial x}+v\frac{\partial u}{\partial y}+w\frac{\partial u}{\partial z}\right)\frac{\partial T}{\partial x}$$

$$+{2\lambda }_{1}\left[uv\frac{{\partial }^{2}T}{\partial x\partial y}+vw\frac{{\partial }^{2}T}{\partial y\partial z}+uw\frac{{\partial }^{2}T}{\partial x\partial z}\frac{\partial T}{\partial x}\right]+{\lambda }_{1}\left(u\frac{\partial v}{\partial x}+v\frac{\partial v}{\partial y}+w\frac{\partial v}{\partial z}\right)\frac{\partial T}{\partial y}$$

$$-\frac{{Q}_{0}{\lambda }_{1}}{(\rho {C}_{p}{)}_{hnf}}\left(u\frac{\partial T}{\partial x}+v\frac{\partial T}{\partial y}+w\frac{\partial T}{\partial z}\right)+{\lambda }_{1}\left(u\frac{\partial w}{\partial x}+v\frac{\partial w}{\partial y}+w\frac{\partial w}{\partial z}\right)\frac{\partial T}{\partial z}$$

6 $$=\frac{{k}_{hnf}}{(\rho {C}_{p}{)}_{hnf}}\frac{{\partial }^{2}T}{{\partial }^{2}y}+\frac{{Q}_{0}}{(\rho {C}_{p}{)}_{hnf}}\left(T-{T}_{\infty }\right),$$

where $u,\,v\,\text{and}\,w \text{are\,the\,velocity\,components},\,T\, \text{is\,the\,temperature},\,C\,\text{is\,the\,concentration},$ ${\rho }_{hnf}$ is the density of the hybrid nanofluid, ${B}_{o}$ is the magnetic field induction, ${\sigma }_{hnf}$ is the electrical conductivity of the hybrid nanofluid, ${\lambda }_{1}$ is the thermal relaxation time, ${k}_{hnf}$ is the thermal conductivity of the hybrid nanofluid, ${\left({c}_{p}\right)}_{hnf}$ is the specific heat in the hybrid nanofluid and ${Q}_{o}$ is the heat generation coefficient.

After implementation of the nonslip mechanism, one can get the boundary conditions which are written below

7 $$\begin{array}{c}u=ax={U}_{w},\hspace{0.33em}v=by={V}_{w},\hspace{0.33em}T={T}_{w},\hspace{0.33em}w=0\hspace{0.33em}{\text{at}}\hspace{0.33em}z=0,\\ v\to 0,\hspace{0.33em}u\to 0,\hspace{0.33em}T\to {T}_{\infty }\hspace{0.33em}{\text{at}}\hspace{0.33em}z\to \infty ,\end{array}$$

The velocity and temperature variables via symmetry analysis are expressed as^44^

8 $$u=ax{f}^{^{\prime}},\hspace{0.33em}v=by{g}^{^{\prime}},\hspace{0.33em}w=-a{\left(\frac{b{a}^{n-2}}{{\rho }_{f}}\right)}^{\frac{1}{n+1}}\left[\frac{2n}{n+1}f+\frac{1-n}{1+n}\eta {f}^{^{\prime}}+g\right]{x}^{\frac{n-1}{n+1}},\theta =\frac{T-{T}_{\infty }}{{T}_{w}-{T}_{\infty }},\eta ={\left(\frac{b{a}^{n-2}}{{\rho }_{f}}\right)}^{\frac{1}{n+1}}z{x}^{\frac{1-n}{1+n}},$$

Above relations for velocity and temperature field help to transform Eqs. (3)–(7) in their dimensionless forms which are

$$ \left( {\left| {f\prime \prime } \right|^{{n - 1}} f^{\prime\prime}} \right)^{\prime }  - (1 - \phi _{2} )\left\{ {(1 - \phi _{1} ) + \phi _{1} \frac{{\rho _{{s_{1} }} }}{{\rho _{f} }}} \right\} + \phi _{2} \frac{{\rho _{{s_{2} }} }}{{\rho _{f} }}\left[ {\left( {f^{\prime}} \right)^{2}  + \left( {\frac{{2n}}{{n + 1}}f + g} \right)f^{\prime\prime}} \right] - \left( {1 - \phi _{1} } \right)^{{2.5}} \left( {1 - \phi _{2} } \right)^{{2.5}} \frac{{\sigma _{{hnf}} }}{{\sigma _{f} }}M^{2} f\prime  = 0, $$

9 $$f(0)=0,\hspace{0.33em}{f}^{^{\prime}}(0)=1,\hspace{0.33em}f(\infty )\to 0,$$

$${\left({\left|{f}^{^{\prime\prime} }\right|}^{n-1}{g}^{^{\prime\prime} }\right)}^{^{\prime}}-(1-{\phi }_{2})\left\{(1-{\phi }_{1})+{\phi }_{1}\frac{{\rho }_{{s}_{1}}}{{\rho }_{f}}\right\}+{\phi }_{2}\frac{{\rho }_{{s}_{2}}}{{\rho }_{f}}\left[({g}^{^{\prime}}{)}^{2}+\left(\frac{2n}{n+1}f+g\right){g}^{^{\prime\prime} }\right]-{\left(1-{\phi }_{1}\right)}^{2.5}{\left(1-{\phi }_{2}\right)}^{2.5}\frac{{\sigma }_{hnf}}{{\sigma }_{f}}{M}^{2}{g}^{^{\prime}}=0,$$

10 $$g(0)=0,\hspace{0.33em}{g}^{^{\prime}}(0)=1,\hspace{0.33em}g(\infty )\to 0,$$

$${\theta }^{^{\prime\prime} }+\frac{{\left(\rho {C}_{p}\right)}_{hnf}{k}_{f}}{{\left(\rho {C}_{p}\right)}_{f}{k}_{hnf}}\left[\mathit{Pr}\left(\frac{2n}{n+1}\right)f{\theta }^{^{\prime}}+\mathit{Pr}g\theta \right]+{\left(\frac{2n}{n+1}f+g\right)}^{2}{\theta }^{^{\prime\prime} }+\frac{{k}_{f}}{{k}_{hnf}}{h}_{s}\left(\frac{2n}{n+1}\right)\mathit{Pr}\theta -\frac{{\left(\rho {C}_{p}\right)}_{hnf}{k}_{f}}{{\left(\rho {C}_{p}\right)}_{f}{k}_{hnf}}\mathit{Pr}{\lambda }_{E}\left[\left(\frac{2n}{n+1}f+g\right)\left(\frac{2n}{n+1}{f}^{^{\prime}}+{g}^{^{\prime}}\right){\theta }^{^{\prime}}\right]+{h}_{s}\mathit{Pr}\left(\frac{2n}{n+1}f{\theta }^{^{\prime}}+g{\theta }^{^{\prime}}\right)=0,$$

11 $$\theta (0)=1,\hspace{0.33em}\theta (\infty )\to 0,$$

The quantities involved in the above equations are expressed^36^ by

$${\rho }_{nf}=(1-\phi ){\rho }_{f}+\varphi {\rho }_{S},\hspace{0.33em}{\rho }_{hnf}=[(1-{\phi }_{2})\{(1-{\phi }_{1}){\rho }_{f}+{\phi }_{1}{\rho }_{{S}_{1}}\}]+{\phi }_{2}{\rho }_{{S}_{2}},$$

$$(\rho {c}_{p}{)}_{nf}=(1-\phi )(\rho {c}_{p}{)}_{f}+\phi (\rho {c}_{p}{)}_{S},\hspace{0.33em}{\mu }_{nf}=\frac{{\mu }_{f}}{(1-\phi {)}^{2.5}},$$

$$(\rho {c}_{p}{)}_{hnf}=[(1-{\phi }_{2})\{\left(1-{\phi }_{1}\right)\left(\rho {c}_{p}{)}_{f}+{\phi }_{1}\left(\rho {c}_{p}{)}_{{S}_{1}}\right\}\right]+{\phi }_{2}(\rho {c}_{p}{)}_{{S}_{2}},$$

$${\mu }_{hnf}=\frac{{\mu }_{f}}{(1-{\phi }_{1}{)}^{2.5}(1-{\phi }_{2}{)}^{2.5}},\frac{{k}_{hnf}}{{k}_{bf}}=\frac{{k}_{{S}_{2}}+(n-1){k}_{bf}-(n-1){\phi }_{2}({k}_{bf}-{k}_{{S}_{2}})}{{k}_{{S}_{2}}+(n-1){k}_{bf}+{\phi }_{2}({k}_{bf}-{k}_{{S}_{2}})},$$

$$\frac{{\sigma }_{nf}}{{\sigma }_{f}}=1+\frac{3(\sigma -1)\phi }{(\sigma +2)-(\sigma -1)\phi },\frac{{\sigma }_{hnf}}{{\sigma }_{bf}}=\frac{{\sigma }_{{S}_{2}}+2{\sigma }_{bf}-2{\phi }_{2}({\sigma }_{bf}-{\sigma }_{{S}_{2}})}{{\sigma }_{{S}_{2}}+2{\sigma }_{bf}+{\phi }_{2}({\sigma }_{bf}-{\sigma }_{{S}_{2}})},$$

12 $$ \frac{{\sigma _{{bf}} }}{{\sigma _{f} }} = \frac{{\sigma _{{S_{1} }}  + 2\sigma _{f}  - 2\phi _{1} (\sigma _{f}  - \sigma _{{S_{1} }} )}}{{\sigma _{{S_{1} }}  + 2\sigma _{f}  + \phi _{1} (\sigma _{f}  - \sigma _{{S_{1} }} )}},\frac{{k_{{nf}} }}{{k_{f} }} = \frac{{k_{S}  + (n - 1)k_{f}  - (n - 1)\phi (k_{f}  - k_{S} )}}{{k_{S}  + (n - 1)k_{f}  + \phi (k_{f}  - k_{S} )}} $$

The dimensionless parameters are ${M}^{2}=\frac{2{\sigma }_{f}{B}_{0}^{2}}{a{\rho }_{f}}$, ${Q}_{h}=\frac{Q}{a({C}_{p}{)}_{f}{\rho }_{f}}$, ${\lambda }_{E}=\frac{a{\lambda }_{1}}{x}$, $\mathit{Re}=\frac{{x}^{n}{\left({U}_{w}\right)}^{2-n}{\rho }_{f}}{{k}_{f}}$ and $\mathit{Pr}=\frac{({C}_{p}{)}_{f}{\rho }_{f}a{x}^{2}{Re}^{\frac{2}{n+1}}}{{k}_{f}}$. These dimensionless parameters, respectively, are called the Hartmann number, the heat generation parameter, the thermal relaxation parameter (thermal Deborah number), the Reynolds number, and the Prandtl number. The subscripts $f,hnf,nf,$ stand for fluid, hybrid nanofluid, and nanofluid respectively and ${s}_{1}$ and ${s}_{2}$ stand for solid particles $Mo{S}_{2}$ and $\mathit{Si}{O}_{2}$ respectively $.$

Stresses (in dimensionless forms) in $x$ and $y$-directions are^44^

13 $$ \begin{gathered}   \frac{1}{2}C_{f} (Re)^{{\frac{1}{{n + 1}}}}  = \frac{1}{{(1 - \phi _{1} )^{{2.5}} (1 - \phi _{2} )^{{2.5}} }}\left| {f^{\prime\prime}\left( 0 \right)} \right|^{n} , \hfill \\   \frac{1}{2}C_{f} (Re)^{{\frac{1}{{n + 1}}}}  = \frac{1}{{(1 - \phi _{1} )^{{2.5}} (1 - \phi _{2} )^{{2.5}} }}\left| {f^{\prime\prime}\left( 0 \right)} \right|^{n} g^{\prime\prime}\left( {\text{0}} \right), \hfill \\  \end{gathered}  $$

The heat transfer rate can be determined through

14 $$Nu=\frac{x{q}_{w}}{{k}_{f}({T}_{w}-{T}_{\infty })},\hspace{0.33em}{q}_{w}=-{k}_{hnf}\frac{\partial T}{\partial z}{|}_{\text{at wall}},\hspace{0.33em}(\mathit{Re}{)}^{-0.5}Nu=-\frac{{k}_{hnf}}{{k}_{f}}{\theta }^{^{\prime}}(0).$$

The numerical values for thermos-physical properties of base fluid and nanoparticles used in the simulations are tabulated in Table 1 given below.

**Table 1:** The numerical values for thermos-physical properties for ${s}_{1}$,${s}_{2}$ and the base fluid.

$\text{Physical properties}$	$\text{Ethylene glycol}$	$Mo{S}_{2}$	$\mathit{Si}{O}_{2}$
$\rho$	$1113.5$	$2650$	$5060$
${c}_{p}$	$2430$	$730$	$397.746$
$k$	$0.613$	$1.5$	$34.5$
$\sigma$	$4.3\times 1{0}^{-5}$	$0.0005$	$1\times 1{0}^{-18}$

## Numerical method

The finite element method (FEM) is the most suitable method for the solution of CFD problems. The convergence associated with FEM can be achieved easily. Further convergence rate of solutions obtained by FEM is faster than other methods like finite volume method, finite difference method, spectral method, etc. The working principle for FEM can consist of the following important steps.

i. Derivation of integral residual statements in their weak forms.
ii. Here, in this investigation, the weak forms are approximated using the Galerkin procedure.
iii. The derivation of stiffness elements and their use in the assembly process. The nonlinear system is obtained via the assembly process.
iv. The nonlinear equations are linearized and obtained linearized system is solved iteratively.
v. Computations are performed to ensure the results to be grid-independent. The grid-independent analysis is done through numerous numerical experiments and outcomes are listed in Table 2. This Table shows that results are independent of mesh size if computational tolerance $[0, 7]$ meshes into zero elements.

**Table 2:** Grid independent results when $\mathit{Pr}=204,\hspace{0.33em}{\lambda }_{E}=0.01,\hspace{0.33em}{h}_{s}=1.3,\hspace{0.33em}n=2,\hspace{0.33em}M=0.001,$ ${\phi }_{1}=0.004,\hspace{0.33em}{\phi }_{2}=0.0075.$

No. of elements	${f}^{^{\prime}}\left(\frac{{\eta }_{\infty }}{2}\right)$	$g^{\prime}\left( {\frac{{\eta _{\infty } }}{{\text{2}}}} \right)$	$\theta \left(\frac{{\eta }_{\infty }}{2}\right)$
30	0.5376460453	0.5376460453	0.5546070594
60	0.5176980663	0.5176980663	0.5377861295
90	0.5115628373	0.5115628373	0.5321685164
120	0.5085855867	0.5085855867	0.5293573362
150	0.5068273585	0.5068273585	0.5276701521
180	0.5056668939	0.5056668939	0.5265445047
210	0.5048435401	0.5048435401	0.5257412993
240	0.5042290630	0.5042290630	0.5251384468
270	0.5037528152	0.5037528152	0.5246705698
300	0.5037531756	0.5033731756	0.5246942273

Equations (9) and (11) are aimed to solve numerically using the FEM. The weighted residual integrals associated with problems (9) and (11) are given by

$${\int }_{{\eta }_{e}}^{{\eta }_{e+1}}{w}_{i}({f}^{^{\prime}}-h)d\eta =0$$

$${\int }_{{\eta }_{e}}^{{\eta }_{e+1}}{w}_{i}({g}^{^{\prime}}-l)d\eta =0$$

$$ \begin{aligned}&{\int }_{{\eta }_{e}}^{{\eta }_{e+1}}{w}_{i}\left[{\left({\left|{\overline{h} }^{^{\prime}}\right|}^{n-1}{h}^{^{\prime}}\right)}^{^{\prime}}-(1-{\phi }_{2})\left\{(1-{\phi }_{1})+{\phi }_{1}\frac{{\rho }_{{s}_{1}}}{{\rho }_{f}}\right\} +{\phi }_{2}\frac{{\rho }_{{s}_{2}}}{{\rho }_{f}}\left[(h)^{2}+\left(\frac{2n}{n+1}\overline{f }+\overline{g }\right){h}^{^{\prime}}\right]\right.\\&\quad\left.-{\left(1-{\phi }_{1}\right)}^{2.5}{\left(1-{\phi }_{2}\right)}^{2.5}\frac{{\sigma }_{hnf}}{{\sigma }_{f}}{M}^{2}h\vphantom{{\left({\left|{\overline{h} }^{^{\prime}}\right|}^{n-1}{h}^{^{\prime}}\right)}^{^{\prime}}-(1-{\phi }_{2})\left\{(1-{\phi }_{1})+{\phi }_{1}\frac{{\rho }_{{s}_{1}}}{{\rho }_{f}}\right\} +{\phi }_{2}\frac{{\rho }_{{s}_{2}}}{{\rho }_{f}}\left[(h)^{2}+\left(\frac{2n}{n+1}\overline{f }+\overline{g }\right){h}^{^{\prime}}\right]}\right]d\eta =0,\end{aligned} $$

$$ \begin{aligned}&{\int }_{{\eta }_{e}}^{{\eta }_{e+1}}{w}_{i}\left[{\left({\left|{\overline{h} }^{^{\prime}}\right|}^{n-1}{l}^{^{\prime}}\right)}^{^{\prime}}-(1-{\phi }_{2})\left\{(1-{\phi }_{1})+{\phi }_{1}\frac{{\rho }_{{s}_{1}}}{{\rho }_{f}}\right\}\right.\\&\quad\left.+{\phi }_{2}\frac{{\rho }_{{s}_{2}}}{{\rho }_{f}}\left[(l{)}^{2}+\left(\frac{2n}{n+1}f+g\right){l}^{^{\prime}}\right]-{\left(1-{\phi }_{1}\right)}^{2.5}{\left(1-{\phi }_{2}\right)}^{2.5}\frac{{\sigma }_{hnf}}{{\sigma }_{f}}{M}^{2}l\vphantom{{\left({\left|{\overline{h} }^{^{\prime}}\right|}^{n-1}{l}^{^{\prime}}\right)}^{^{\prime}}-(1-{\phi }_{2})\left\{(1-{\phi }_{1})+{\phi }_{1}\frac{{\rho }_{{s}_{1}}}{{\rho }_{f}}\right\}}\right]d\eta =0,\end{aligned} $$

$${\int }_{{\eta }_{e}}^{{\eta }_{e+1}}{w}_{i}\left[{\theta }^{^{\prime\prime} }+\frac{{\left(\rho {C}_{p}\right)}_{hnf}{k}_{f}}{{\left(\rho {C}_{p}\right)}_{f}{k}_{hnf}}\left[\mathit{Pr}\left(\frac{2n}{n+1}\right)f{\theta }^{^{\prime}}+\mathit{Pr}g\theta \right]+{\left(\frac{2n}{n+1}f+g\right)}^{2}{\theta }^{^{\prime\prime} }+\frac{{k}_{f}}{{k}_{hnf}}{h}_{s}\left(\frac{2n}{n+1}\right)\mathit{Pr}\theta -\frac{{\left(\rho {C}_{p}\right)}_{hnf}{k}_{f}}{{\left(\rho {C}_{p}\right)}_{f}{k}_{hnf}}\mathit{Pr}{\lambda }_{E}\left(\frac{2n}{n+1}f+g\right)\left(\frac{2n}{n+1}h+l\right){\theta }^{^{\prime}}+{h}_{s}\mathit{Pr}\left(\frac{2n}{n+1}f{\theta }^{^{\prime}}+g{\theta }^{^{\prime}}\right)\right]d\eta =0,$$

where ${f}^{^{\prime}}=h and {g}^{^{\prime}}=l$. $\text{weight\,functions} ({w}_{2},{w}_{3}$, and ${w}_{4}$). ${\eta }_{e}$ and ${\eta }_{e+1}$ are nodes of typical element $e$. The unknown dependent variables $f,\hspace{0.33em}h,$ and $\theta$ are approximated by expansions: $f={\sum }_{j=1}^{2}{f}_{j}{\psi }_{j},$ $\theta ={\sum }_{j=1}^{2}{\theta }_{j}{\psi }_{j}$ and $h={\sum }_{j=1}^{2}{h}_{j}{\psi }_{j}$, where ${f}_{j},\hspace{0.33em}{\theta }_{j},$ and ${h}_{j},$ are the nodal values. ${w}_{j}={\psi }_{j}$ are the linear shape function.

Using the above approximations in the weak formulation of weighted residuals, the stiffness, and the boundary elements are given by

$${K}_{ij}^{11}={\int }_{{\eta }_{e}}^{{\eta }_{e+1}}{\psi }_{i}\frac{d{\psi }_{j}}{d\eta }d\eta , {K}_{ij}^{12}=-{\int }_{{\eta }_{e}}^{{\eta }_{e+1}}{\psi }_{i}{\psi }_{j}d\eta ,$$

$${K}_{ij}^{13}={K}_{ij}^{14}={K}_{ij}^{15}=0$$

$${K}_{ij}^{21}={K}_{ij}^{23}={K}_{ij}^{24}={K}_{ij}^{25}=0,$$

$${K}_{ij}^{22}={\int }_{{\eta }_{e}}^{{\eta }_{e+1}}{w}_{i}\left[\left({\left|{\overline{h} }^{^{\prime}}\right|}^{n-1}\right){\psi }_{i}\frac{d{\psi }_{j}}{d\eta }-(1-{\phi }_{2})\left\{(1-{\phi }_{1})+{\phi }_{1}\frac{{\rho }_{{s}_{1}}}{{\rho }_{f}}\right\}+{\phi }_{2}\frac{{\rho }_{{s}_{2}}}{{\rho }_{f}}\left[h{\psi }_{i}{\psi }_{j}+\left(\frac{2n}{n+1}\overline{f }+\overline{g }\right){\psi }_{i}\frac{d{\psi }_{j}}{d\eta }\right]-{\left(1-{\phi }_{1}\right)}^{2.5}{\left(1-{\phi }_{2}\right)}^{2.5}\frac{{\sigma }_{hnf}}{{\sigma }_{f}}{M}^{2}{\psi }_{i}{\psi }_{j}\right]d\eta $$

$${K}_{ij}^{31}={K}_{ij}^{32}={K}_{ij}^{35}=0$$

$${K}_{ij}^{33}={\int }_{{\eta }_{e}}^{{\eta }_{e+1}}{\psi }_{i}\frac{d{\psi }_{j}}{d\eta }d\eta , {K}_{ij}^{34}=-{\int }_{{\eta }_{e}}^{{\eta }_{e+1}}{\psi }_{i}{\psi }_{j}d\eta ,$$

$${K}_{ij}^{41}={K}_{ij}^{42}={K}_{ij}^{45}=0$$

$${K}_{ij}^{44}={\int }_{{\eta }_{e}}^{{\eta }_{e+1}}\left[{\left|{\overline{h} }^{^{\prime}}\right|}^{n-1}{\psi }_{i}\frac{d{\psi }_{j}}{d\eta }-\left(1-{\phi }_{2}\right)\left\{\left(1-{\phi }_{1}\right)+{\phi }_{1}\frac{{\rho }_{{s}_{1}}}{{\rho }_{f}}\right\}+{\phi }_{2}\frac{{\rho }_{{s}_{2}}}{{\rho }_{f}}\overline{l}{\psi  }_{i}{\psi }_{j}+{\phi }_{2}\frac{{\rho }_{{s}_{2}}}{{\rho }_{f}}\left[+\left(\frac{2n}{n+1}\overline{f }+\overline{g }\right){\psi }_{i}\frac{d{\psi }_{j}}{d\eta }\right]-{\left(1-{\phi }_{1}\right)}^{2.5}{\left(1-{\phi }_{2}\right)}^{2.5}\frac{{\sigma }_{hnf}}{{\sigma }_{f}}{M}^{2}{\psi }_{i}{\psi }_{j}\right]d\eta ,$$

$${K}_{ij}^{51}={K}_{ij}^{52}={K}_{ij}^{54}={K}_{ij}^{54}=0,$$

$${K}_{ij}^{55}={\int }_{{\eta }_{e}}^{{\eta }_{e+1}}\left[-\frac{d{\psi }_{i}}{d\eta }\frac{d{\psi }_{j}}{d\eta }+\frac{{\left(\rho {C}_{p}\right)}_{hnf}{k}_{f}}{{\left(\rho {C}_{p}\right)}_{f}{k}_{hnf}}\left[\mathit{Pr}\left(\frac{2n}{n+1}\right)\overline{f}{\psi  }_{i}\frac{d{\psi }_{j}}{d\eta }+\mathit{Pr}\overline{g}{\psi  }_{i}{\psi }_{j}\right]-{\left(\frac{2n}{n+1}\overline{f }+\overline{g }\right)}^{2}\frac{d{\psi }_{i}}{d\eta }\frac{d{\psi }_{j}}{d\eta }+\frac{{k}_{f}}{{k}_{hnf}}{h}_{s}\left(\frac{2n}{n+1}\right)\mathit{Pr}{\psi }_{i}{\psi }_{j}+{h}_{s}\mathit{Pr}\left(\frac{2n}{n+1}\overline{f}{\psi  }_{i}\frac{d{\psi }_{j}}{d\eta }+\overline{g}{\psi  }_{i}\frac{d{\psi }_{j}}{d\eta }\right)-\frac{{\left(\rho {C}_{p}\right)}_{hnf}{k}_{f}}{{\left(\rho {C}_{p}\right)}_{f}{k}_{hnf}}\mathit{Pr}{\lambda }_{E}\left[\left(\frac{2n}{n+1}\overline{f }+\overline{g }\right)\left(\frac{2n}{n+1}\overline{h }+\overline{l }\right){\psi }_{i}\frac{d{\psi }_{j}}{d\eta }\right]\right]d\eta ,$$

where $\overline{{f_{i} }} ,\;\overline{{g_{i} }} ,\;\overline{{l_{i} }} ,\;\overline{{h_{i} }} ,$ and $\bar{\theta }_{i}$ are nodal values computed at the previous iteration.

*Assembly procedure* we get the system of nonlinear algebraic equations of the form

$$[K\{\pi \}]\{\pi \}=\{F\},$$

where $[K\{\pi \}]$ is the global stiffness matrix.

*Stiffness matrix* It is important to note that the stiffness matrix $[K\{\pi \}]$ is also a function of unknown nodal values. Therefore, a system of algebraic equations is be solved numerically by an iterative procedure. Here in this study, the Picard linearization procedure is used which works in the following way

$$[K\{\pi {\}}^{r-1}]\{\pi {\}}^{r}=\{F\}$$

where $\{\pi {\}}^{r-1}$ are nodal values computed at $(r-1{)}{th}$ iteration and $\{\pi {\}}^{r}$ are the nodal values being computed at the ${r}{th}$ iteration.

*Domain* The computational domain is $[0, 7].$

*Error* The error in the simulated results is calculated using

$$error=\left|{\pi }^{r}-{\pi }^{r-1}\right|$$

till convergence criteria

$$max\left|{\pi }_{i}^{r}-{\pi }_{i}^{r-1}\right|<\varepsilon =1{0}^{-5}$$

is satisfied.

The numerical values for density specific heats, thermal conductivities, and electrical conductivities for solid particles ${s}_{1}$, ${s}_{2}$ and the base fluid are tabulated in Table 2 given below.

It is very important to mention that no experimental study related to the present work is available. Moreover, a theoretical study on the three-dimensional flow of power-law fluid with the numerical data as a special case of present work is not available in the literature. Therefore, it is not possible to provide validation of our results. However, the results found by two different methods are compared and their comparison is presented in Table 3.

**Table 3:** The comparison of results for $Mo{S}_{2}/\mathit{Si}{O}_{2}$-nanofluid using two methods (homotopy analysis method and the finite element method) when $M=0.001,{\lambda }_{E}=,0.3{h}_{s}=0.3$ and $Sc\; = 0.3$ $\mathit{Pr}=204,\hspace{0.33em}{h}_{s}=1.3,\hspace{0.33em}{\phi }_{1}=0.004,\hspace{0.33em}{\phi }_{2}=0.0075.$

		Homotopy analysis method			The finite element method		
		$-(\mathit{Re}{)}^{-\frac{1}{2}}{C}_{f}$	$-(\mathit{Re}{)}^{-\frac{1}{2}}{C}_{g}$	$(\mathit{Re}{)}^{-\frac{1}{2}}Nu$	$-(\mathit{Re}{)}^{-\frac{1}{2}}{C}_{f}$	$-(\mathit{Re}{)}^{-\frac{1}{2}}{C}_{g}$	$(\mathit{Re}{)}^{-\frac{1}{2}}Nu$
$n$	1	2.19048887	2.69435000	3.40917100	2.19048867	2.69435020	3.40917113
	2	2.26914900	2.69506080	3.40830920	2.26914914	2.69506077	3.40830920
	3	2.36832100	2.71301081	3.74498558	2.36832103	2.71301077	3.74498557

## Results and discussion

Rheological models, models of hybrid nanoparticles, and fundamental dimensionless equations are solved numerically using FEM. After, ensuring convergence, grid independence, and validation of results, numerical experiments for the behaviors of related parameters on field variables are done. The simulations are visualized and recorded in graphical and numerical data. Following is the discussion.

### Velocity components and variation of parameters

**Figure 2:**
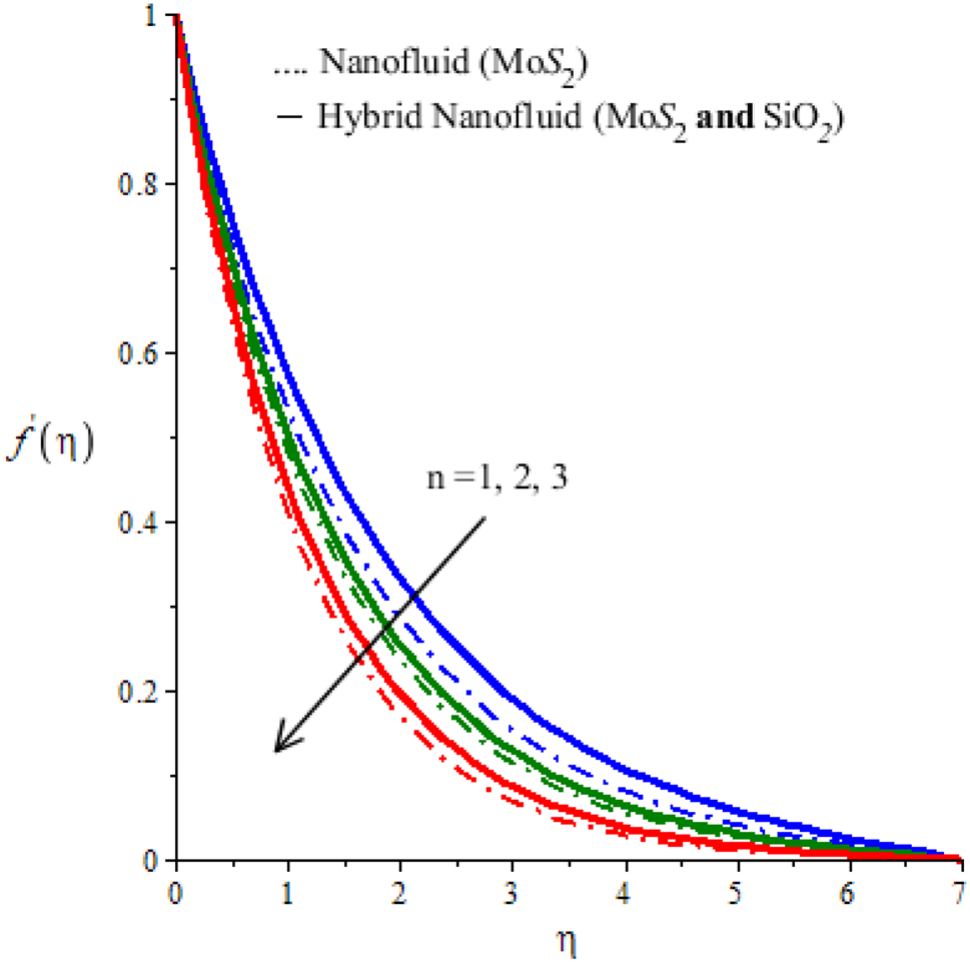
Horizontal velocity profiles of mono nanofluid (dotted curves) and hybrid nanofluid (solid curves) for $n$ when $\mathit{Pr}=204,\hspace{0.33em}{\lambda }_{E}=0.01,\hspace{0.33em}{h}_{s}=1.3,\hspace{0.33em}M=0.001,\hspace{0.33em}{\phi }_{1}=0.004,\hspace{0.33em}{\phi }_{2}=0.0075.$

**Figure 3:**
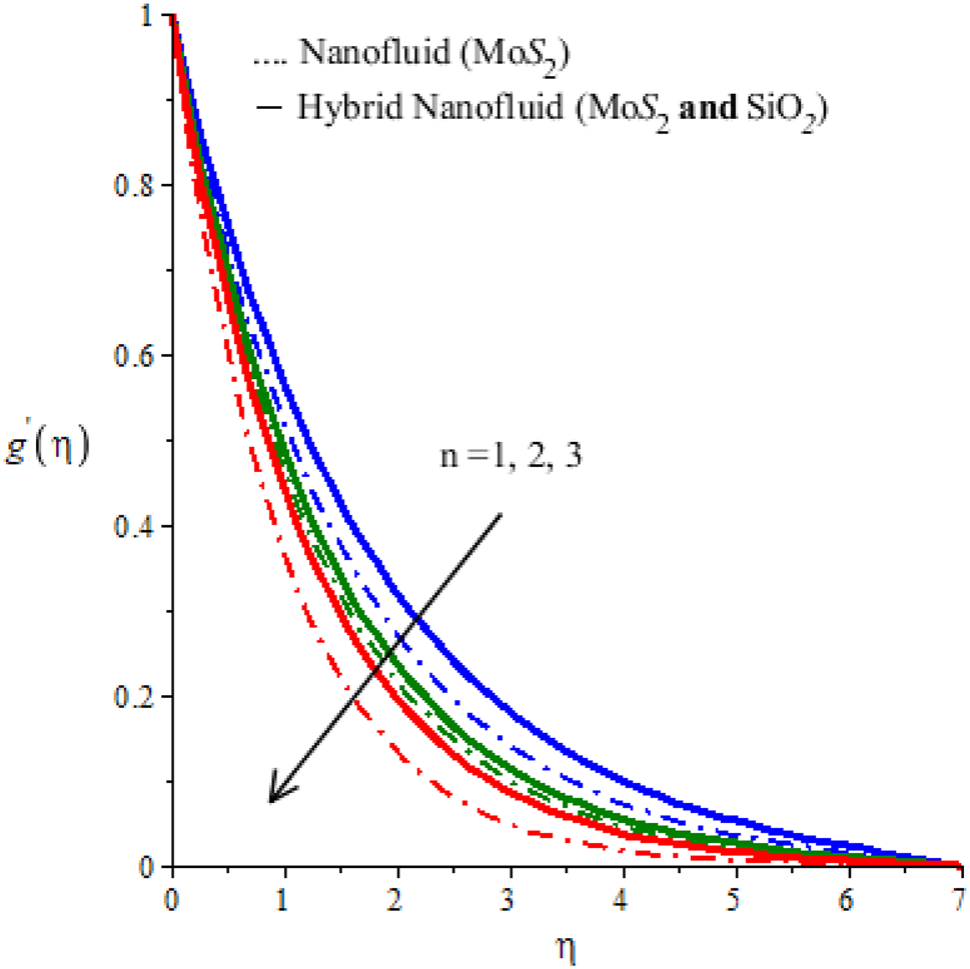
Vertical velocity profiles of mono nanofluid (dotted curves) and hybrid nanofluid (solid curves) for $n$ when $\mathit{Pr}=204,\hspace{0.33em}{\lambda }_{E}=0.2,\hspace{0.33em}{h}_{s}=1.5,\hspace{0.33em}M=0.01,\hspace{0.33em}{\phi }_{1}=0.004,\hspace{0.33em}{\phi }_{2}=0.0075.$

**Figure 4:**
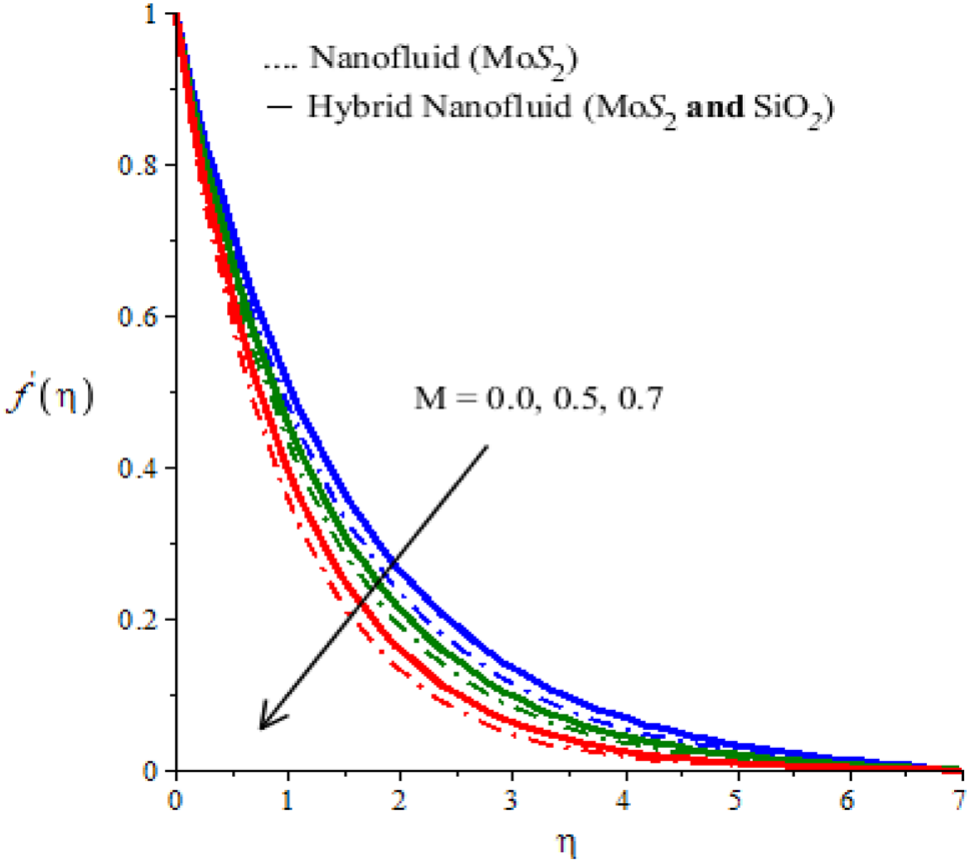
Horizontal velocity profiles of mono nanofluid (dotted curves) and hybrid nanofluid (solid curves) for $M$ when $\mathit{Pr}=204,\hspace{0.33em}{\lambda }_{E}=0.01,\hspace{0.33em}{h}_{s}=1.3,\hspace{0.33em}n=2,\hspace{0.33em}{\phi }_{1}=0.004,\hspace{0.33em}{\phi }_{2}=0.0075.$

**Figure 5:**
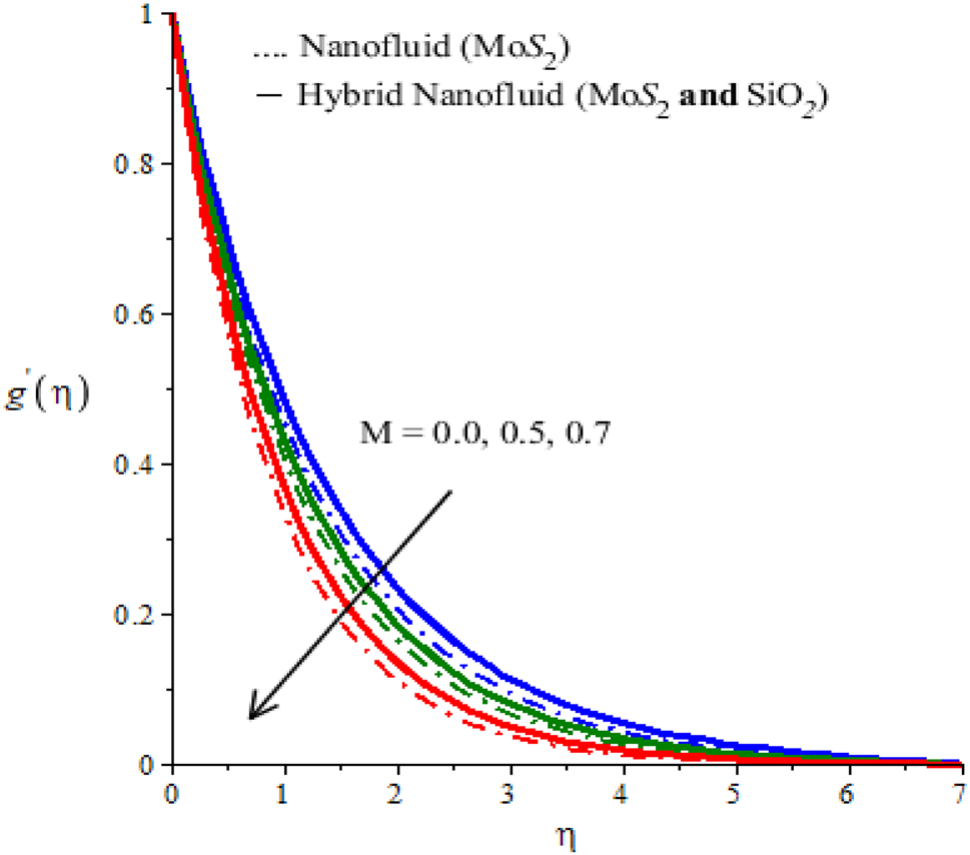
Vertical velocity profiles of mono nanofluid (dotted curves) and hybrid nanofluid (solid curves) for $M$ when $\mathit{Pr}=204,\hspace{0.33em}{\lambda }_{E}=0.5,\hspace{0.33em}{h}_{s}=1.7,\hspace{0.33em}n=2,\hspace{0.33em}{\phi }_{1}=0.004,\hspace{0.33em}{\phi }_{2}=0.0075.$

The parameter $n$ appears in the constitutive equation for fluid is called power-law fluid. For $n=1$ the modeled, equations reduce to the case of a Newtonian fluid. The case $n>1$ is called the shear thickening case. The behavior of n on $x$ and $y$ components of velocity is examined and observed outcomes are displayed in Figs. 2 and 3. These Figures predict that both components of velocity have decreasing tendency for values of n greater than 1. However, this declination in the case of $Mo{S}_{2}-\mathit{Si}{O}_{2}$-power-law fluid is greater than that for $Mo{S}_{2}$-power-law fluid. Thus viscous region for the Newtonian fluid is wider than the viscous region for nano-power law fluid (see Figs. 2 and 3). Figures 4 and 5 determine the behavior of velocity components against the variation of Hartmann number $M$. An increase in $M$ is because of an increase in the intensity of the magnetic field which is responsible for an increase in the magnitude of opposing force. Consequently, the flow of fluid slows down. This decreasing tendency of motion of fluid particles is observed in both $x$ and $y$ -directions. Thus it is concluded that the width of the viscous region can be controllable via an applied magnetic field. Figures 4 and 5 also demonstrate that the Lorentz force induced due to the motion of $Mo{S}_{2}-\mathit{Si}{O}_{2}$-power-law fluid is weaker than the Lorentz force induced due to the motion of $Mo{S}_{2}$-power-law fluid. Alternatively, boundary layer thickness for $Mo{S}_{2}$-power-law fluid is greater than the boundary layer thickness for of $Mo{S}_{2}-\mathit{Si}{O}_{2}$-power-law fluid.

### Temperature field and variation of related parameters

**Figure 6:**
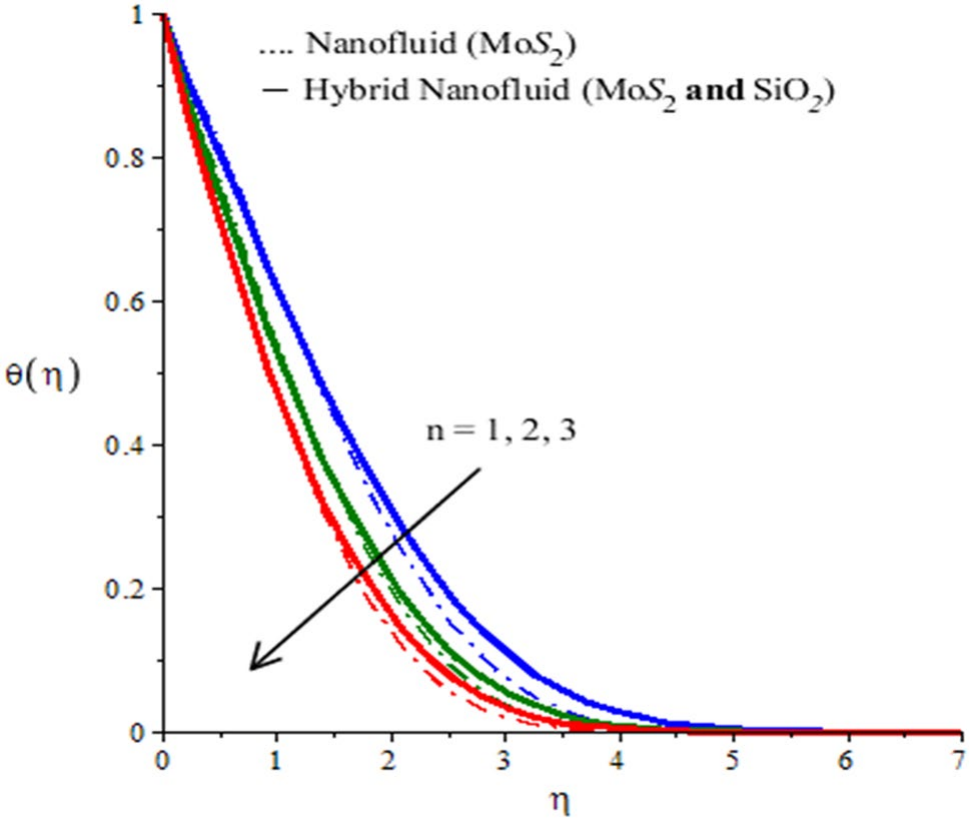
Temperature profiles of mono nanofluid (dotted curves) and hybrid nanofluid (solid curves) for $Ec$ when $\mathit{Pr}=204,\hspace{0.33em}{\lambda }_{E}=0.1,\hspace{0.33em}{h}_{s}=1.3,\hspace{0.33em}M=0.7,\hspace{0.33em}{\phi }_{1}=0.004,\hspace{0.33em}{\phi }_{2}=0.0075.$

**Figure 7:**
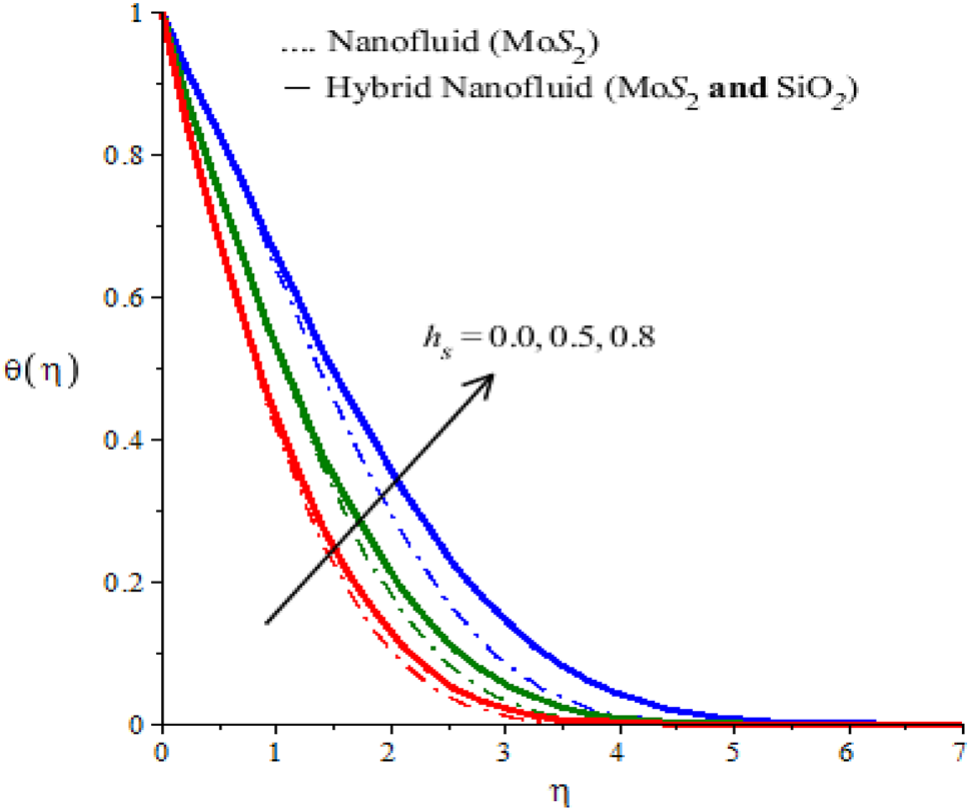
Temperature profiles of mono nanofluid (dotted curves) and hybrid nanofluid (solid curves) for ${h}_{s}$ when $\mathit{Pr}=204,\hspace{0.33em}{\lambda }_{E}=2.0,\hspace{0.33em}n=2,\hspace{0.33em}M=0.001,\hspace{0.33em}{\phi }_{1}=0.004,\hspace{0.33em}{\phi }_{2}=0.0075.$

**Figure 8:**
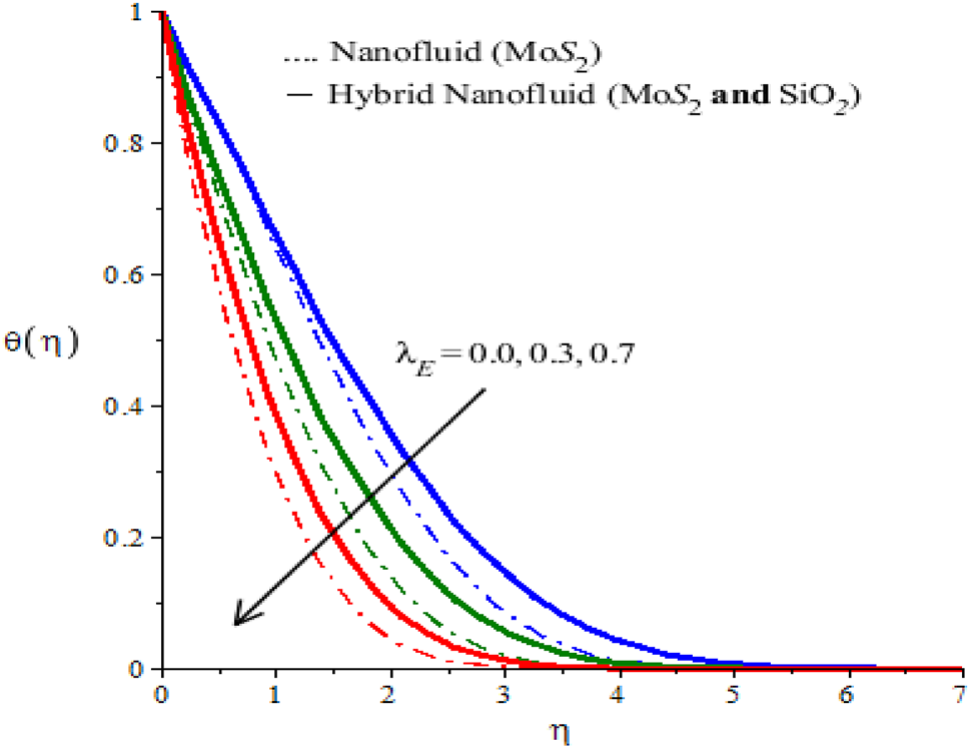
Temperature profiles of mono nanofluid (dotted curves) and hybrid nanofluid (solid curves) for ${\lambda }_{E}$ when $\mathit{Pr}=204,\hspace{0.33em}{\lambda }_{E}=2.4,\hspace{0.33em}{h}_{s}=1.7,\hspace{0.33em}n=2,\hspace{0.33em}M=0.001,\hspace{0.33em}{\phi }_{1}=0.004,\hspace{0.33em}{\phi }_{2}=0.0075.$

The behaviors of parameters $n$,${h}_{s}$ and ${\lambda }_{E}$ on the temperature, the field is visualized through numerical simulations and observed behaviors are recorded in the form of graphs given by Figs. 6, 7 and 8 for both types of fluids ($Mo{S}_{2}-\mathit{Si}{O}_{2}$-power-law fluid and $Mo{S}_{2}$-power-law fluid). Figure 6 demonstrates a decrease in the temperature versus rheological power index $n$ appearing in the rheological equation. Since the variation of $n$ through positive values result in a decrease in the flow. Therefore, the convective transfer of heat is compromised. This compromise of convective heat transfer leads to a decrease in the temperature (see Fig. 6). Moreover, the thermal region shrunk when $n$ is increased. The parameter ${h}_{s}$ is called the heat generation parameter and it determines the impact of the ability of the fluid to generate heat. This generated heat adds to the fluid to increase the temperature. Thus increasing the behavior of ${h}_{s}$ on the temperature of the fluid can be seen from Fig. 7. This Figure also reflects that the hybrid nanofluid is more heat generative than the mono nanofluid. The role of thermal relaxation time on heat transfer can be seen in Fig. 8. A decline in temperature against thermal relaxation time is the ability of the fluid to restore its thermal equilibrium state. Therefore, an increase in thermal relaxation parameter ${\lambda }_{E}$ causes the thermal changes to be minimized. Consequently, temperature decreases.

The above discussion has revealed that if ethylene glycol with hybrid nanostructures has been used as engine coolant, its cooling performance is much better than pure ethylene glycol or ethylene with $Mo{S}_{2}$. It also noticed that heat generation may affect the cooling performance of $Mo{S}_{2}-\mathit{Si}{O}_{2}-$ ethylene glycol as it generates more heat relative to pure the ethylene and $Mo{S}_{2}-$ ethylene glycol. This is a disadvantage of $Mo{S}_{2}-\mathit{Si}{O}_{2}-$ ethylene glycol towards its used as a coolant. Thus it is recommended that the base fluid must be non-heat generating otherwise, its thermal performance will be compromised. This is a limitation toward its usage as a coolant. Consequently, the cooling performance of the hybrid nanofluid (here, in this case, $Mo{S}_{2}-\mathit{Si}{O}_{2}-$ ethylene glycol) will be improved. These characteristics of power-law fluid may counter heat generation. Further, the thermal boundary layer thickness is noticed to be controlled by the thermal relaxation parameter. In many boundary layer flow passing over the surface like the surface of aircraft, automobile vehicles. etc., Lorentz force for the case when ethylene glycol does not exhibit Ohmic dissipation helps control the thermal boundary layer thickness. However, in the case of Ohmic dissipated fluid, Lorentz force in controlling the thermal boundary layer is less effective. This is another limitation of the working fluid.

### Discussion about behaviors of tangential stresses and wall heat transfer rate

The behavior of tangential stresses and heat flux are examined for sampling values of $$n,$$$$M,$$ $${\lambda }_{E}$$ and $${h}_{s}$$ for both $$Mo{S}_{2}-\mathit{Si}{O}_{2}$$-power-law fluid and $$Mo{S}_{2}$$-power-law fluid. The parameter $$n$$ appears in the rheological model designed for power-law fluids and its variation (for positive values greater than $$1$$) corresponds to the case of shear thickening whereas $$n$$ has valued less than 1 for the shear-thinning case. Shear thickening behavior of fluid makes it able to experience less wall influence and therefore, shear rate dependent viscosity decreases, and as a result wall momentum penetrates fluid slowly. Due to this fact, tangential stresses at the surface in both $$x$$ and $$y$$ -directions become stronger. This observation is valid for both fluids ($$Mo{S}_{2}-\mathit{Si}{O}_{2}$$-power-law and $$Mo{S}_{2}$$-power-law). Numerical experiments have demonstrated an increase in heat transfer rate against increasing values of $$n$$ greater than 1. The parameter $$M$$ is called the Hartmann number and its variation determines the variation of the intensity of the magnetic field. Since Lorentz force is directly proportional to the intensity of the magnetic field, therefore, flow experiences retardation due to an increase in the intensity of the magnetic field. Alternatively, one can say that an increase in $$M$$ implies an increase in retardation towards flow. The increase in tangential stresses in the $$x$$ and $$y$$-direction is noted. Further motion due to the Lorentz force slows down and therefore, convective transport of heat is compromised. Thus convective transport of heat will be reduced and heat flux will decrease. The numerical simulations have predicted the same results (see Table 4). Thermal relaxation parameter $${\lambda }_{E}$$ also has a great impact on wall heat flux and therefore its behavior on heat transfer rate is examined and outcomes are displayed in Table 4. The numerical values tabulated in Table 4 show that wall heat flux for $$Mo{S}_{2}-\mathit{Si}{O}_{2}$$-power-law fluid and $$Mo{S}_{2}$$-power-law fluid has shown decreasing behavior versus $${\lambda }_{E}$$. The heat generation parameter has also decreasing behavior on the wall heat transfer rate.

**Table 4:** Numerical values of velocity gradient, heat and mass fluxes versus $n,M,{\lambda }_{E},{K}_{s}$ and $Sc$ when $\mathit{Pr}=204,\hspace{0.33em}{h}_{s}=1.3,\hspace{0.33em}{\phi }_{1}=0.004,\hspace{0.33em}{\phi }_{2}=0.0075.$

		$Mo{S}_{2}$-nanofluid			$Mo{S}_{2}/\mathit{Si}{O}_{2}$-nanofluid		
		$-(\mathit{Re}{)}^{-\frac{1}{2}}{C}_{f}$	$-(\mathit{Re}{)}^{-\frac{1}{2}}{C}_{g}$	$(\mathit{Re}{)}^{-\frac{1}{2}}Nu$	$-(\mathit{Re}{)}^{-\frac{1}{2}}{C}_{f}$	$-(\mathit{Re}{)}^{-\frac{1}{2}}{C}_{g}$	$(\mathit{Re}{)}^{-\frac{1}{2}}Nu$
$n$	1	0.15300384	0.32732733	0.0773821	2.19048867	2.69435020	3.40917113
	2	0.29988378	0.47127954	1.8780056	2.26914914	2.69506077	3.40830920
	3	0.34549393	0.48800919	2.1978578	2.36832103	2.71301077	3.74498551
$M$	0.0	0.44453571	0.54742391	2.4342852			2.52199998
	0.3	0.52762208	0.61610260	2.3670662			2.43935049
	0.7	0.70658376	0.78080389	2.2013857	0.16254512	0.39211179	0.45823071
${\lambda }_{E}$	0.0	0.82741445	0.78609554	1.4898677	0.51652670	0.27875076	0.23835808
	0.7	0.95774385	0.81920321	2.4532322	0.62699791	0.83176414	6.19927907
	1.3	0.98698227	0.85111488	2.4149498	0.68019279	0.90244938	5.27826836
${h}_{s}$	0.0	0.42767763	0.47289141	3.0366932	0.36994133	0.56727947	8.07024755
	0.3	0.42767763	0.47289141	2.2654646	0.36994133	0.56727947	7.11929662
	0.7	0.42767763	0.47289141	2.1894274	0.36994133	0.56727947	6.16243262

## Conclusion

Governing laws in terms of differential equations associated with a thermal enhancement in ethylene glycol due to the dispersion of $Mo{S}_{2}$ and combination of $Mo{S}_{2}$ and ${SiO}_{2}$ are solved numerically by FEM. Several numerical experiments were performed. The following results are notable.

- The comparative analysis between thermal efficiencies of $Mo{S}_{2}$-power-law fluid and $Mo{S}_{2}-\mathit{Si}{O}_{2}$-power-law fluid is presented. It is found that the thermal efficiency of power-law fluid with $Mo{S}_{2}$ and $\mathit{Si}{O}_{2}the$ nanostructures are greater than that of the power-law fluid with only $Mo{S}_{2}$ nanoparticles. It is also noted that pure power-law fluid has less thermal conductivity than that of $Mo{S}_{2}$-power-law fluid and $Mo{S}_{2}-\mathit{Si}{O}_{2}$-power-law fluid.
- The heat generation may affect the cooling performance by $Mo{S}_{2}-\mathit{Si}{O}_{2}-$ ethylene glycol as it generates more heat relative to pure ethylene glycol and $Mo{S}_{2}-$ ethylene glycol. This is a disadvantage of $Mo{S}_{2}-\mathit{Si}{O}_{2}-$ ethylene glycol towards its used as a coolant. Thus it is recommended that base fluid must be non-heat generating otherwise performance by $Mo{S}_{2}-\mathit{Si}{O}_{2}-$ ethylene glycol will be compromised.
- It is recommended to use ethylene glycol with hybrid nanostructures as an engine coolant, its cooling performance is much better than pure ethylene glycol or ethylene with $Mo{S}_{2}.$
- In many boundaries layer flow passing over the surface like the surface of aircraft, automobile vehicles, etc. Lorentz force for the case when ethylene glycol does not exhibit Ohmic dissipation. However, in the case of Ohmic dissipated fluid, Lorentz force in controlling the thermal boundary layer is less effective. This is another limitation of the working fluid.
- Non-Fourier heat transfer is slower than Fourier transfer due to thermal memory effects based on thermal relaxation time. Thus, thermal changes tend to be restored due to the thermal relaxation phenomenon.
- The power-law fluid with nanoparticles ($Mo{S}_{2}$ and $\mathit{Si}{O}_{2}$) is assumed to heat generative. The numerical experiments for comparison between heat generative rate in $Mo{S}_{2}$-power-law fluid and heat generative rate in $Mo{S}_{2}-\mathit{Si}{O}_{2}$-power-law fluid are performed and it is observed that power-law fluid with hybrid nanoparticles is more heat generative than a power-law fluid with mono-nanoparticles ($Mo{S}_{2}$). Thus it can also be concluded that pure power-law fluid is less heat generative than a power-law fluid with nanoparticles. Thus if the power-law fluid has to be used as a coolant then if it should be non-heat generative. On the other hand, a power-law fluid with nanoparticles serves as a stable coolant. Therefore, the power-law fluid serves as a better coolant if it is non-heat generative.
- It is found that thermal relaxation time for power-law fluid with $Mo{S}_{2}-\mathit{Si}{O}_{2}$ nanoparticles are lesser than that for power-law fluid with $Mo{S}_{2}$ nanoparticles. Thus power-law fluid with $Mo{S}_{2}$ and $\mathit{Si}{O}_{2}$ is more capable to restore thermal changes relative to power-law fluid with $Mo{S}_{2}$ nanoparticles only.
- Magnetic field is responsible for inducing Lorentz force which is responsible for creating shear stresses on the surface. Thus tangential stresses are increased by increasing the magnetic field intensity.
- Heat flux decreases when the heat generative parameter is increased.
- There is a significant difference between heat fluxes for $Mo{S}_{2}$-power-law fluid and $Mo{S}_{2}-\mathit{Si}{O}_{2}$-power-law fluid. Thus the use of $Mo{S}_{2}-\mathit{Si}{O}_{2}$-power-law fluid is recommended as it has much greater heat flux than that of $Mo{S}_{2}$-power-law fluid.

### Future perspective

In literature, ethylene glycol has not been proved or disproved to be heat generating. If it is heat generating then obviously its thermal performance will be compromised. Contrary to this if ethylene glycol is not a heated generative then it is a stable coolant and its thermal performance will be optimized. The present era is a time of new and innovative technologies. Thus manufacturing of non-heat generating fluid will be possible in coming days.
